# Lithography-Free Technology for the Preparation of Digital Microfluidic (DMF) Lab-Chips with Droplet Actuation by Optoelectrowetting (OEW)

**DOI:** 10.1155/2022/2011170

**Published:** 2022-05-29

**Authors:** Christoph Doering, Johannes Strassner, Henning Fouckhardt

**Affiliations:** Integrated Optoelectronics and Microoptics Research Group, Physics Department, Technische Universität Kaiserslautern (TUK), PO Box 3049, D-67653, Kaiserslautern, Germany

## Abstract

Electrically conducting liquid droplets can be activated and moved by electrowetting-on-dielectric (EWOD) and optoelectrowetting (OEW). An important application is droplet manipulation in digital microfluidics (DMF, lab-on-a-chip 2.0) as a chip-sized chemical laboratory. For spectroscopic analyses of chemical reactions, it is often necessary to prepare or examine the reagent droplets before, during, and after the reaction. With OEW, single droplets with volumes of 50–250 nl can be moved, analyzed, and merged in one pipetting step. To ensure analysis sensitivity in many applications, lab-chips should only be used once due to contamination of the surface and chemical modification of the layers by the droplets. Single-use chip preparation without a lithographic step, e.g., for the definition of the spacer layer, reduces efforts and costs. Here, exemplarily, we demonstrate the OEW-driven movement and mixing of chemical reagents in a simple color change reaction analyzed by absorption spectroscopy. Stripes made from the insulating tape serve as spacers between sub and superstrate, and any lithographic step can be avoided.

## 1. Introduction

Analytical chemistry has experienced dramatic changes during the last three decades. For specific tasks, formerly, lab-filling instrumentation could be reduced in size to lab-on-a-chip (version 1.0) [1]. The analyte solutions are stored in microvials and flowing through capillary channels on the chip, e.g., pumped electrophoretically [2]. This way, much smaller fluid volumes both for the analytes as well as the solvents are possible. Moreover, due to miniaturization and ease of automation, separation procedures have become faster, more reproducible, and thus more reliable. Meanwhile, at least for certain tasks, another transition has taken place, i.e., the one to droplet or so-called digital microfluidics (DMF) or labs-on-a-chip 2.0. The analyte solutions are electrically conductive nano or picoliter droplets—not flowing, but rather resting or moving on top of a substrate [2–8]. Such concepts are especially helpful wherever very small volumes of the analyte are available, e.g., the fluid amount of only a couple of living cells.

In DMF, the droplets have to be moved on the lab-chip to certain positions, where specific analytical tasks have to be pursued, e.g., intermixing or chemical reactions [9–12]. Oftentimes, the individual spectroscopic fingerprint of each reagent and reaction product has to be determined, e.g., by infrared spectroscopy [13], surface-enhanced Raman scattering (SERS) [14–17], or fluorescence detection [18], to name just a few methods.

Optoelectrowetting [19–24] is based on electrowetting-on-dielectric (EWOD), where the droplet and the layer sequence of the chip form a capacitor, and loading of this capacitor reduces surface tension and the contact angle of the droplet on the dielectric layer [21–23]. If the droplet is initially only partially sitting above the structured bottom electrode layer, the change will be local, and the droplet will be pulled onto the electrode. EWOD and spectroscopical detection like SERS [17] or absorption spectroscopy can be combined.

In the case of optoelectrowetting, an additional photoconductive layer beneath the dielectric one is used. A typically perpendicularly impinging light beam locally changes the capacitance and the interplay of the complex-valued impedances of the layers. Thus, the operating point of the device can be optimized by change of voltage and of its modulation frequency. The main advantage of OEW as compared to EWOD is that the bottom electrode does not have to be structured—the droplet is moved, wherever the light spot is moved to—which makes the production process much simpler.

For DMF applications, possibly, perpendicular incidence of the light beam might constitute a restriction because the related optical components block the space beneath or above the lab-chip. Thus, in [25, 26], we have presented a concept, called light line OEW (LL-OEW), where the light beam is traversing the chip surface parallel from side to side.

In EWOD as well as OEW, for droplet actuation, usually, a superstrate is used, i.e., the chip is not only consisting of the substrate and its layers on top but also of a superstrate with the top electrode layer beneath in order to contact the droplets electrically, no matter where they are on the substrate. Beyond this purpose, the superstrate also reduces droplet evaporation considerably, an otherwise severe problem in DMF.

**Figure 1:**
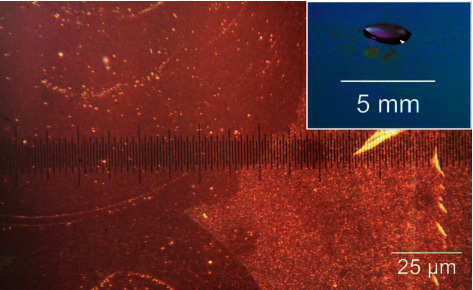
Photographs showing that the analyte and solvent might chemically react with the layer sequence, thus prohibiting the multiple use of the chip. Wherever droplets have touched the layers upon their actuation, surface modifications have taken place. The photograph in the inset shows a larger area of the chip under oblique observation. The droplet has been moved slightly away from the position, where it sat during an analytical run. Modified surface spots are revealed.

Substrate and superstrates are usually separated by and bonded via a spacer layer. The voids in the spacer layer also define volumes in which the droplets can be moved. Although the layers directly on the substrate do not have to be structured for OEW and, thus, do not require lithographic technology, the spacer layer has to be structured lithographically and makes the production process more complicated and expensive. This will be even more of a problem, if the analytes or reagents chemically attack the layer sequence (possibly in combination with the high electrical fields) because, in this case, the chips can only be used once, and the costs of their production should be reduced as much as possible.  Figure 1 shows how droplet contact can deteriorate layer quality. Especially, spectroscopic analyses can be disturbed.

In this contribution, we introduce a lithography-free and, thus, a much simpler technological process to prepare lab-chips for DMF applications. To give an example for a chemical reaction on a DMF chip with OEW droplet actuation, we choose a simple color change reaction of sodium hydroxide (NaOH) and phenolphthalein (C_20_H_14_O_4_), which is used innumerably in chemistry, but here in combination with absorption spectroscopy. In principle, the set-up is very similar, e.g., to a SERS set-up [17]. There, the Raman pump light is impinging onto the chip from one side; here, it is the background illumination. There, the scattered light is detected on the same or the other side of the chip; here, it is the remaining transmitted light after absorption in the droplets. And here, the droplets are actuated by OEW with a perpendicularly impinging laser light beam, which can also be done in SERS applications.

## 2. Chip and Analytical Concept

### 2.1. Principle Layer/Chip Design

**Figure 2:**
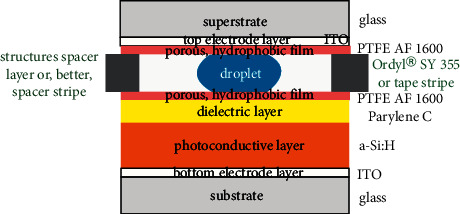
Cross-sectional view (not to scale) of layer sequence of DMF chip. Typical materials and layer thicknesses from bottom to top: 1.0 mm thick glass slide (substrate), (100 ± 30) nm thick ITO electrode layer, (780 ± 10) nm thick a-Si : H photoconductive layer, (360 ± 10) nm thick Parylene C dielectric layer, approx. 10 nm thin hydrophobic PTFE AF 1600 film, from 50 *μ*m up to 250 *μ*m thick spacer stripe or 100 *μ*m thick insulating tape, approx. 10 nm thick hydrophobic PTFE AF 1600 film again, (100 ± 30) nm thick ITO electrode layer, and 1.0 mm thick glass slide (superstrate).

Figure 2 shows a cross-sectional view of a typical chip design. Since, in general, optical access to the droplets should be possible for reasons of OEW, and some analytical measurement techniques, the substrate, the superstrate, and the electrode layers are made from transparent materials. Substrates and superstrates are borosilicate glass slides. The electrode layers are made from a transparent oxide, namely, indium tin oxide (ITO). Actually, complete ITO-coated glass slides from Merck KGaA, Darmstadt, Germany, are used.

SÜSS MicroTec directly above and below the droplet, there are 10 nm thick hydrophobic layers, made of polytetrafluoroethylene, i.e., PTFE AF 1600 from Sigma Aldrich, Taufkirchen, Germany, spin-coated using a Delta BM Gyrset from SÜSS Microtech, Garching, Germany. Since they are thin and porous, the electrical contacts of the droplet to the dielectric layer as well as to the top electrode layer are guaranteed. The PTFE layers are intended to allow for larger initial contact angles of the droplets and contact angle changes by voltage or control light. In the case of a water droplet, the initial contact angle is about 120° in our case.

Typically, a (360 ± 10) nm thick dielectric layer made of Parylene C is employed; its raw material is from Specialty Coating System Inc., Indianapolis, IN, USA. It is deposited by chemical vapor deposition using a PDS 2010 system from Specialty Coating System Inc., Indianapolis, IN, USA. Hydrogenated amorphous silicon (a-Si : H) is used for the (790 ± 10) nm thick photoconductive layer and deposited onto the ITO-coated bottom glass slide by plasma-enhanced chemical vapor deposition (PE-CVD) with a PlasmaLab 80 Plus machine from Oxford Instruments Plasma Technology, Bristol, UK.

The layer most important in the context of this contribution is the spacer between the substrate and the superstrate part of the chip/module. Previously [25, 26], we have used Ordyl® SY 355 for this purpose. It is a dry film resist from ElgaEurope s.r.l, Milano, Italy. The thickness of a single Ordyl® SY 355 layer is 50 *μ*m. The dry film resist has been applied to the substrate by hot-roll lamination of two to five layers (giving a total thickness of 100*–*250 *μ*m). It has been structured photolithographically. The number of lamination steps determines the spacing between substrate and superstrate parts of the module. This has to be chosen such that a certain range of droplet volumes can be used with the chip because the droplet has to touch both the top electrode layer (with PTFE coating) as well as the dielectric layer (with PTFE coating).

The lamination, together with the lithographical step, makes the technological process time-consuming and expensive, even more thinking of single chip use. Thus, we switched from the lithographically structured Ordyl® SY 355 spacer layer to stripes made from insulating tape. The material is polyvinyl chloride (PVC), and the tape is available in various thicknesses from 50 to 150 *μ*m, thus providing for a range of droplet volumes approximately between 50 and 250 nl.

### 2.2. Choice of the Simple Test for Demonstration

One of the greatest challenges of lab-on-chip 1.0 technology has been the detection of minute amounts of substances in hair-thin channels. While a wide range of detection methods has been developed for more conventional lab-on-a-chip systems, there is a great need for research in this area in the context of digital microfluidics (DMF, lab-on-a-chip 2.0). For this purpose, almost exclusively electrochemical approaches or fluorescence detection have been reported in the literature [7–9]. Although fluorescence microscopy offers impressive possibilities for visualizing droplet motion, it is subjected to the obvious limitation that only very few molecules show fluorescence. But DMF is open for virtually any chemical analysis technique. To name another example beyond infrared spectroscopy, Raman scattering, and fluorescence detection, the coupling of a DMF chip with mass spectrometry has been presented by Wheeler's group [27].

Especially for organic chemical syntheses, the identification of intermediate and final products on the chip will be necessary. Thus, the development and application of detection methods for lab-on-a-chip 2.0 technology is the main focus of current research.

Here, just for demonstration, as a very simple but innumerably often used example for a chemical reaction, we employ a color change reaction for pH indication in combination with absorption spectroscopy.

### 2.3. Optical Set-Up, Mounting, and Electrical Activation

**Figure 3:**
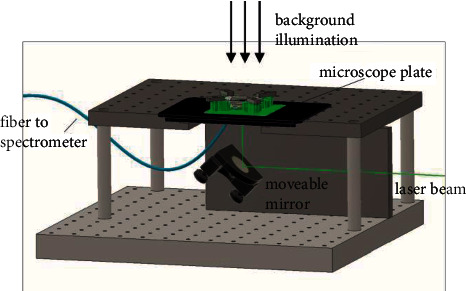
Drawing of the analytical set-up. On the left is a microscope plate with the chip holder to be described in Figure 4. A fiber of a spectrometer is attached underneath. The OEW light from a laser diode at a wavelength of 520 nm is regulated with a neutral density filter to a power of 150 nW and enters the set-up from the bottom directed via moveable mirrors.

The transmission of the individual droplets can also be measured before the reaction. We observed the color change of sodium hydroxide solution on the indicator phenolphthalein. The transmission of the set-up background illumination through the OEW-chip has been measured as well as the transmission of the droplets before and after the reaction. For this purpose, the droplets have been moved on the chip by OEW with the help of a laser beam impinging perpendicularly from below the module, as shown in Figure 3.

The illumination from the bottom is provided with a laser diode L520P50 from Thorlabs GmbH, Bergkirchen, Germany The laser diode has an emission wavelength of *λ* = 520 nm. The intensity of the beam is 0.74 W/m^2^ due to a reduction with a neutral density filter and the elliptical cross-section and spot with semiaxes of 0.75 and 0.27 mm. The transmission spectra are measured with a fiber spectrometer USB-2000 from Ocean Insight, Duiven, the Netherlands. Background illumination is used. On the left side of the set-up in Figure 3, a microscope could be placed, as it is used for many analytical investigations (FT-IR microscopy and Raman microscopy). Here, only a microscope plate is used. The perpendicular fiber tip is located on the bottom of the microscope table plate. The laser light comes from the right and is directed onto the chip via mirrors.

**Figure 4:**
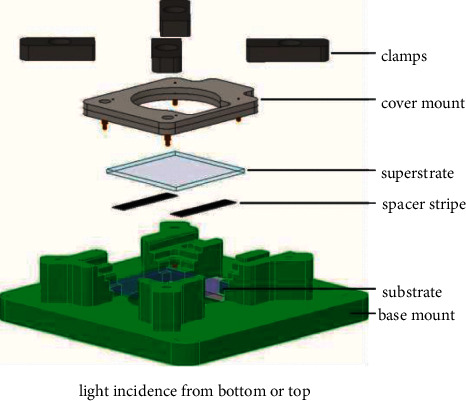
Exploded assembly drawing of set-up with lab-chip parts. The OEW light is entering the set-up from the bottom (possibly also from the top). The part, which is drawn green, is designed to be clipped into a commonly used microscope table. Substrate and superstrates (with their respective layers) are pressed into this mount separated by stripes of insulating the PVC tape. The whole assembly is joined mechanically by a cover mount (shown greyish here) and four clamps.

The bottom and top parts of the module are joined by spring-loaded pins 0906-1-15-20-75-14-11-0 from Mill-Max Manufacturing Corp., Oyster Bay, NY, USA.  Figure 4 shows an exploded view of the assembly and the chip. The green drawn sample holder is 3D-printed and adapted to a commonly used microscope table as part of many typical spectroscopical set-ups. Both parts of the module are pressed into this mount, separated by stripes of insulating the PVC tape. The whole assembly is joined mechanically by a cover mount (shown greyish here) and four clamps.

**Figure 5:**
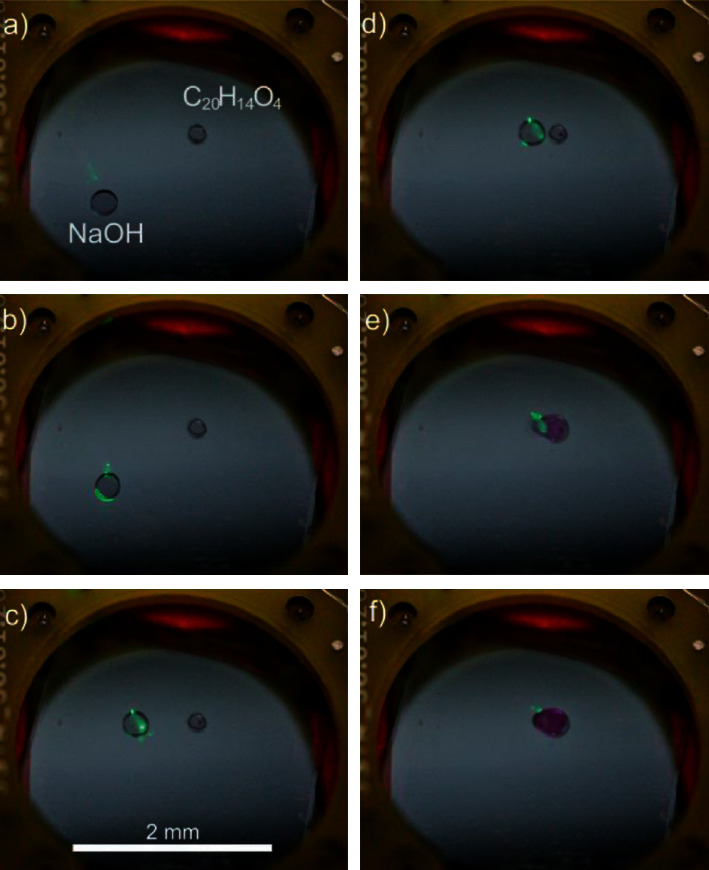
Frames from a video showing the droplet movement and mixing of sodium hydroxide (NaOH) and phenolphthalein (C_20_H_14_O_4_) with the help of laser beam-based OEW. The beam is perpendicularly incident from below the chip. A spot of scattered light can be observed, since the photoconductive a-Si : H layer is thin enough (780 nm) to transmit some of the OEW control light at 520 nm wavelength. The NaOH droplet is moved to the C_20_H_14_O_4_ droplet and both merged. The new droplet changes the color from colorless to purple. Transmission spectra of the single droplets and the resulting droplet are measured.

A rectangular alternating voltage in the range of ± (25…60) V (for the video frames in Figure 5 ±27 V) at a frequency of 50 Hz is applied.

## 3. Droplets and Droplet Movement

For the matter of demonstration, the reagents sodium hydroxide (NaOH) in a concentration of 250 mM and phenolphthalein (C_20_H_14_O_4_) in a concentration of 37 mM are diluted in deionized water (5 *μ*S/cm). The droplets of the different reagents are deposited on the superstrate with a micropipette with a volume of 50–150 nl.

Side remark: another principle possibility for droplet injection, which should be favored for automated analyses, could be provided by a part of the lab-chip, which contains capillary fluid channels, which forward amounts of liquids from microvials. Thus, lab-on-a-chip 1.0 technology can be used to support the lab-on-a-chip 2.0 or DMF technology, as suggested by Belder et al. [17].

Figure 5 shows frames from a video showing droplet movement with the help of the mentioned laser beam spot and merging of the reagents. The sodium hydroxide droplet is moved to the position of the other droplet. The mixing and the corresponding color change reaction elapse within three video frames (*t* < 3 1/24 *s* = 1/8 s).

**Figure 6:**
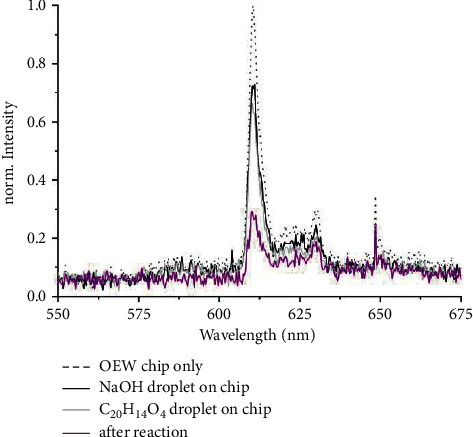
Transmission spectra of the OEW-chip without droplets (black dotted), of the chip with sodium hydroxide droplet (black line), of the chip with the phenolphthalein droplet (gray line), and after reaction of phenolphthalein and sodium hydroxide (purple line).

The transmission spectra of the droplets measured with the fiber spectrometer are shown in Figure 6. The remaining light intensity after passage through the initially colorless phenolphthalein (C_20_H_14_O_4_) droplet amounts to about 70% of the intensity remaining after traversing just the module (with substrate and superstrate and their layers). Upon the color change reaction, the transmission drops by another factor of 2.6 or 4.1 dB.

## 4. Conclusions

Digital microfluidics (DMF) for analytical chemistry on microdroplets (lab-on-a-chip 2.0 technology) is a promising field of research and development for many analytical applications, where amounts of used analytes and solvents should be cut down or where only minute sample volumes are available. Yet, DMF should be made viable for everyday work in the lab.

First, this includes opportunities for actuation of droplets on the lab-chip. Second, a cost-effective technology is mandatory, especially in cases when the lab-chip can only be used once due to detrimental chemical interaction with the analytes or solvents.

Here, demonstrated by absorption spectroscopy as a prominent analytical measurement example, the first aspect can be accomplished by optoelectrowetting (OEW). The second goal is achieved by avoiding lithographical steps in the technological process for chip preparation. Substrate and superstrates do not have to be bonded permanently. A temporary bond will suffice oftentimes. In our case, this bond is achieved by pressing sub and superstrates with their respective layers onto each other and into amount, which, e.g., can be inserted into a microscope set-up. Upon clamping, the separation between the substrate and the superstrate part of the chip/module can be accomplished by an object as simple as a stripe of a plastic material. In our case, it has to be electrically insulating to allow for the use of droplet actuation by OEW. Thus, we have employed insulating polyvinyl chloride (PVC) tape.

## Data Availability

The data used to support the findings of this study are available from the corresponding author upon reasonable request. A video to support Figure 5 is within the Supplementary Materials.

## Supplementary Materials

Supplementary Materials A video to Figure 5 is given as Supplementry Material or can be found under the following Internet link: https://vcm.uni-kl.de/Panopto/Pages/Viewer.aspx?id=246733c6-cb14-4314-a3a2-aea200ce0a18

## References

[1] Manz A., Graber N., Widmer H. M. (1990). Miniaturized total chemical analysis systems: a novel concept for chemical sensing. *Sensors and Actuators B: Chemical*.

[2] Hardt S., Schoenfeld F. (2007). *Microfluidic Technologies for Miniaturized Analysis Systems*.

[3] Li P. C. H. (2005). *Microfluidic Lab-on-a-Chip for Chemical and Biological Analysis and Discovery*.

[4] Fouillet Y., Jary D., Chabrol C., Claustre P., Peponnet C. (2007). Digital microfluidic design and optimization of classic and new fluidic functions for lab on a chip systems. *Microfluidics and Nanofluidics*.

[5] Fair R. B. (2007). Digital microfluidics: is a true lab-on-a-chip possible?. *Microfluidics and Nanofluidics*.

[6] Day P., Manz A., Zhang Y. (2012). *Microdroplet Technology: Principles and Emerging Applications in Biology and Chemistry*.

[7] Barber R. W., Emerson D. W., Day P., Manz A., Zhang Y. (2012). Recent advances in electrowetting microdroplet technologies,” Chap. 4. *Microdroplet Technology—Principles and Emerging Applications in Biology and Chemistry*.

[8] Cao J., Schneider S., Schultheiß R., Schober A., Kohler J. M., Groß G. A. (2015). From microtiter plates to droplets—tools for micro-fluidic droplet processing. *Microsystem Technologies*.

[9] Cho S., Moon H., Kim C. (2003). Creating, transporting, cutting, and merging liquid droplets by electrowetting-based actuation for digital microfluidic circuits. *Journal of Microelectromechanical Systems*.

[10] Pei S. N., Wu M. C. On-chip blade for accurate splitting of droplets in light-actuated digital microfluidics.

[11] Shenderov A. D., Pollack M. G. (2012). Methods of dispensing and withdrawing liquid in an electrowetting device.

[12] Jagath N. Y., Nikapitiya B., You S. M., Moon H. Droplet dispensing and splitting by electrowetting on dielectric digital microfluidics.

[13] Pasquini C. (2018). Near infrared spectroscopy: a mature analytical technique with new perspectives—a review. *Analytica Chimica Acta*.

[14] Benz C., Retzbach H., Nagl S., Belder D. (2013). Protein-protein interaction analysis in single microfluidic droplets using FRET and fluorescence lifetime detection. *Lab on a Chip*.

[15] Meier T. A., Poehler E., Kemper F. (2015). Fast electrically assisted regeneration of on chip SERS substrates. *Lab on a Chip*.

[16] Meier T. A., Beulig R. J., Klinge E., Fuss M., Ohla S., Belder D. (2015). On-chip monitoring of chemical syntheses in microdroplets via surface-enhanced Raman spectroscopy. *Chemical Communications*.

[17] Panneerselvam R., Sadat H., Höhn E.-M., Das A., Noothalapati H., Belder D. (2022). Microfluidics and surface-enhanced Raman spectroscopy, a win–win combination?. *Lab on a Chip*.

[18] Sultangaziyev A., Bukasov R. (2020). Review: applications of surface-enhanced fluorescence (SEF) spectroscopy in bio-detection and biosensing. *Sensing and Bio-Sensing Research*.

[19] Grier D. G. (2003). A revolution in optical manipulation. *Nature*.

[20] Chiou P.-Y., Moon H., Toshiyoshi H., Kim C.-J., Wu M. C. (2003). Light actuation of liquid by optoelectrowetting. *Sensors and Actuators A: Physical*.

[21] Chiou P.-Y., Chang Z., Wu M. C. (2008). Droplet manipulation with light on optoelectrowetting device. *Journal of Microelectromechanical Systems*.

[22] Chiou P.-Y., Park S.-Y., Wu M. C. (2008). Continuous optoelectrowetting for picoliter droplet manipulation. *Applied Physics Letters*.

[23] Collier C. M., Hill K. A., DeWachter M. A., Huizing A. M., Holzman J. F. (2015). Nanophotonic implementation of optoelectrowetting for microdroplet actuation. *Journal of Biomedical Optics*.

[24] Findenegg G. H., Herminghaus S. (1997). Wetting: statics and dynamics. *Current Opinion in Colloid and Interface Science*.

[25] Doering C., Strassner J., Fouckhardt H. (2021). Microdroplet actuation via light line optoelectrowetting (LL-OEW). *International Journal of Analytical Chemistry*.

[26] Strassner J., Doering C., Oliveira E., Fouckhardt H. (2022). Optoelectrowetting (OEW) with push-actuation of microdroplets at small frequencies and OEW equations revisited. *Sensors and Actuators A: Physical*.

[27] Choi K., Ng A. H. C., Fobel R., Wheeler A. R. (2012). Digital microfluidics. *Annual Review of Analytical Chemistry*.

