# Supplementary material for: Lithography-Free Technology for the Preparation of Digital Microfluidic (DMF) Lab-Chips with Droplet Actuation by Optoelectrowetting (OEW)
Source: Int J Anal Chem. 2022 May 29;2022:2011170. doi: 10.1155/2022/2011170 (PMC9201745; doi:10.1155/2022/2011170)
Supplement: Supplementary Materials — A video to Figure 5 is given as Supplementry Material or can be found under the following Internet link: https://vcm.uni-kl.de/Panopto/Pages/Viewer.aspx?id=246733c6-cb14-4314-a3a2-aea200ce0a18 [file 2011170.f1.docx]

**Supplementary Materials**

A video to Fig. 5 can be found under the following internet link: https://seafile.rlp.net/f/0be5bfd2adda47909d15/
